# Editorial: Molecular -genetic causes underlying primary adrenal insufficiency: Current insights into diagnosis and treatment

**DOI:** 10.3389/fendo.2022.995151

**Published:** 2022-08-29

**Authors:** Maria Candida B.V. Fragoso, Tânia A. S. S. Bachega, Liliana Dain

**Affiliations:** ^1^ Unidade de Adrenal, Disciplina de Endocrinologia, Laboratório de Hormônios e Genética Molecular (LIM 42), Hospital das Clínicas, São Paulo University, São Paulo, Brazil; ^2^ Buenos Aires University, Departamento de Diagnóstico Genético, Centro Nacional de Genética Médica- ANLIS, Buenos Aires, Argentina; ^3^ Facultad de Ciencias Exactas y Naturales, Universidad de Buenos Aires, Buenos Aires, Argentina

**Keywords:** adrenal insufficiency, management and treatments of adrenal insufficiency, etiology and genetic causes, diagnosis, molecular mechanism

The loss of adrenal cortex function leads to glucocorticoid and/or mineralocorticoid deficiency, ranging from mild nonspecific symptoms to life-threatening shock conditions. Adrenal insufficiency (AI) is classified as primary, secondary or tertiary when the disease results from disorders affecting the adrenal cortex, anterior pituitary or hypothalamus, respectively. In newborns and children, genetic factors are the most frequent causes of AI, with emphasis on congenital adrenal hyperplasia, while acquired etiologies are more frequent in adults.

The diagnosis of all forms of AI is usually delayed because the initial presentation is often non-specific; despite significant advances in knowledge over the last decade, the diagnosis and management of adrenal insufficiency still represent a challenge for physicians, researchers, and also for patients. Moreover, the presence of genetic conditions resulting in AI is often underestimated in clinical practice and, consequently, leads to significant impairment of patients’ quality of life. A relevant point is the need of special attention to patients with latent AI in order to prevent an adrenal crisis during stress conditions. Several studies have showed an increased morbidity and mortality rate in AI patients; therefore, prevention is of fundamental importance. The continuous education of both medical teams and patients/relatives on AI and the management of adrenal crisis is necessary to improve clinical outcomes. Recent studies have focused on developing new types of steroid replacements to mimic the rhythm of cortisol secretion and function, as well as to decrease the metabolic adverse outcomes related to long-term therapy. Further advances in steroid replacements, oral and parenteral, will probably emerge in the coming years.

In this regard, in this issue Younes et al., presented a comprehensive review of the etiologies, diagnosis, and treatments of chronic and acute Primary AI. Management of AI in times of COVID-19 outbreak was also addressed in a promising article of Sabaddin et al., as patients are facing their primary disease and the risk/fear of COVID-19 infection. An especial attention was focused in this issue to Congenital Adrenal Hyperplasia (CAH) a group of autosomal recessive enzymatic disorders, caused by a deficiency of one of the enzymes required for cortisol biosynthesis in the adrenal cortex. CAH secondary to 21-hydroxylase deficiency is the most common form of CAH. Carrozza et al., Arriba et al., Kocova et al., and Marino et al.,outstanding reviewed the current state of the art of this deficiency and its related condition CAH-X (CAH and Ehlers-Danlos Syndrome), ranging from the genetic and the molecular complexity of the locus, to its molecular diagnosis and management. Kim et al., drew attention to the increased prevalence of cardiometabolic risk factors in patients with CAH, claiming that a better understanding of the traditional and non-traditional risk factors in youth with CAH could help guide treatment options and prevent the onset of metabolic syndrome in adulthood, reducing overall patient morbidity. In addition, Ladjouze et al., added their experience in Algeria with the 3β-hydroxysteroid dehydrogenase type 2 deficiency, a rare cause of CAH with an estimated birth prevalence less than 1/1.000.000 and with fewer than 200 families reported worldwide.

Congenital disorders affecting adrenal function may also be associated with diseases of sex development as Finkielstain et al., addressed it in an excellent review article in this topic. Finally, Teoli et al., also described the impact of *NR0B1* (*DAX1*) genetic variants on clinical, hormonal, histological, spermiological aspects and gonadotropin treatment response in male patients with X-linked adrenal hypoplasia congenita (X-AHC).

To conclude, the purpose of this Research Topic was to compile in a single issue the most recent state of the art and new insights on AI, bringing together a comprehensive compendium of etiologies, diagnosis and treatments of the different AI disorders, based on the excellent contributions of the expert authors in the area.

## Author contributions

MF, TB, and LD contributed to the conceptualization and writing of the Editorial. All authors contributed to the article and approved the submitted version.

## Conflict of interest

The authors declare that the research was conducted in the absence of any commercial or financial relationships that could be construed as a potential conflict of interest.

## Publisher’s note

All claims expressed in this article are solely those of the authors and do not necessarily represent those of their affiliated organizations, or those of the publisher, the editors and the reviewers. Any product that may be evaluated in this article, or claim that may be made by its manufacturer, is not guaranteed or endorsed by the publisher.

